# Frequency and risk factors for major complications after stereotactic radiofrequency ablation of liver tumors in 1235 ablation sessions: a 15-year experience

**DOI:** 10.1007/s00330-020-07409-0

**Published:** 2020-10-30

**Authors:** Peter Schullian, Edward Johnston, Gregor Laimer, Daniel Putzer, Gernot Eberle, Arno Amann, Maria Effenberger, Manuel Maglione, Martin C. Freund, Alexander Loizides, Reto Bale

**Affiliations:** 1 Section of Interventional Oncology - Microinvasive Therapy (SIP), Department of Radiology, Medical University of Innsbruck, Anichstr. 35, 6020 Innsbruck, Austria; 2 The Royal Marsden Hospital, 203 Fulham Road, Chelsea, London, SW3 6JJ UK; 3 Department of Internal Medicine V, Medical University of Innsbruck, Anichstr. 35, 6020 Innsbruck, Austria; 4 Department of Internal Medicine I, Medical University of Innsbruck, Anichstr. 35, 6020 Innsbruck, Austria; 5 Department of Visceral, Transplant and Thoracic Surgery, Center of Operative Medicine, Medical University of Innsbruck, Anichstr. 35, 6020 Innsbruck, Austria; 6 Department of Radiology, Medical University of Innsbruck, Anichstr. 35, 6020 Innsbruck, Austria

**Keywords:** Radiofrequency ablation, Liver, Neoplasm, Therapy

## Abstract

**Objectives:**

To assess the frequency of major complications after multi-probe stereotactic radiofrequency ablation (SRFA) in a large cohort of patients over 15 years and to elucidate risk factors for adverse events.

**Materials and methods:**

A retrospective study was carried out between July 2003 and December 2018. Seven hundred ninety-three consecutive patients (median 65.0 years (0.3–88), 241 women and 552 men, were treated in 1235 SRFA sessions for 2475 primary and metastatic liver tumors with a median tumor size of 3.0 cm (0.5–18 cm). The frequency of major complications was evaluated according to SIR guidelines and putative predictors of adverse events analyzed using simple and multivariable logistic regression.

**Results:**

Thirty-day mortality after SRFA was 0.5% (6/1235) with an overall major complication rate of 7.4% (91/1235). The major complication rate decreased from 11.5% (36/314) (before January 2011) to 6.0% (55/921) (*p* = 0.001). 50.5% (46/91) of major complications were successfully treated in the same anesthetic session by angiographic coiling for hemorrhage and chest tube insertion for pneumothorax. History of bile duct surgery/intervention, number of coaxial needles, and location of tumors in segment IVa or VIII were independent prognostic factors for major complications following multivariable logistic regression analysis. Simple logistic regression revealed the number of tumors, tumor size, location close to the diaphragm, tumor conglomerate, and segment VII as other significant predictors.

**Conclusion:**

SRFA of liver tumors is safe and can extend the treatment spectrum of conventional RFA. Adaptations over time combined with increasing experience resulted in a significant decrease in complications.

**Key Points:**

• *In 1235 ablation sessions in 793 patients over 15 years, we found a mortality rate of 0.5% (6/1235) and an overall major complication rate of 7.4%, which fell from 11.5 (36/314) to 6.0% (55/921, p = 0.001) after January 2011, likely due to procedural adaptations.*

• *History of bile duct surgery/intervention (p = 0.013, OR = 3.290), number of coaxial needles (p = 0.026, OR = 1.052), and location of tumors in segment IVa (p = 0.016, OR = 1.989) or VIII (p = 0.038, OR = 1.635) were found to be independent prognostic factors.*

• *Simple logistic regression revealed that number of tumors, tumor size, location close to the diaphragm, tumor conglomerates, and segment VII were other significant predictors of major complications.*

## Introduction

Hepatic resection remains a major strategy in the management of both primary and metastatic liver tumors, although it is associated with morbidity rates of 14–55% [1–3]. More recently, percutaneous radiofrequency ablation (RFA) has become widely accepted as a less invasive treatment strategy in the management of small unresectable liver tumors including hepatocellular carcinoma (HCC) and liver metastases from primaries including colorectal, breast, and neuroendocrine [4–6]. RFA has shown comparable overall survival after hepatic resection in patients with very-early-stage HCC [7] and demonstrates lower morbidity varying from 0.9 to 10.0% [4, 8, 9]. However, the technique necessitates a safety margin whereby the ablation zone completely surrounds the tumor by 10 mm to achieve local tumor control and favorable clinical outcome [10–12]. Achieving adequate treatment margins may be challenging using US or CT guidance alone, particularly for large and irregularly shaped tumors or in situations when the target tumor is either difficult to visualize, awkward to access, or adjacent to vulnerable structures. Moreover, challenging locations pose a higher risk of complications where stereotactic guidance is not used [13, 14]. Stereotaxy is already widely used in radiotherapy and neurosurgery and facilitates the execution of pre-defined three-dimensional plans to enable more complex and difficult ablations. For example, tumors high in the hepatic dome or caudate lobe require steep and often long routes, where three-dimensional planning and precision targeting allow transthoracic routes to be avoided with optimal distribution of RF probes around the target tumor.

The purpose of the current study is to assess the frequency of major complications after multi-probe SRFA with intraoperative image fusion, to evaluate adaptations over time and determine risk factors accounting for adverse events.

## Materials and methods

### Patient cohort and inclusion criteria

**Fig. 1:**
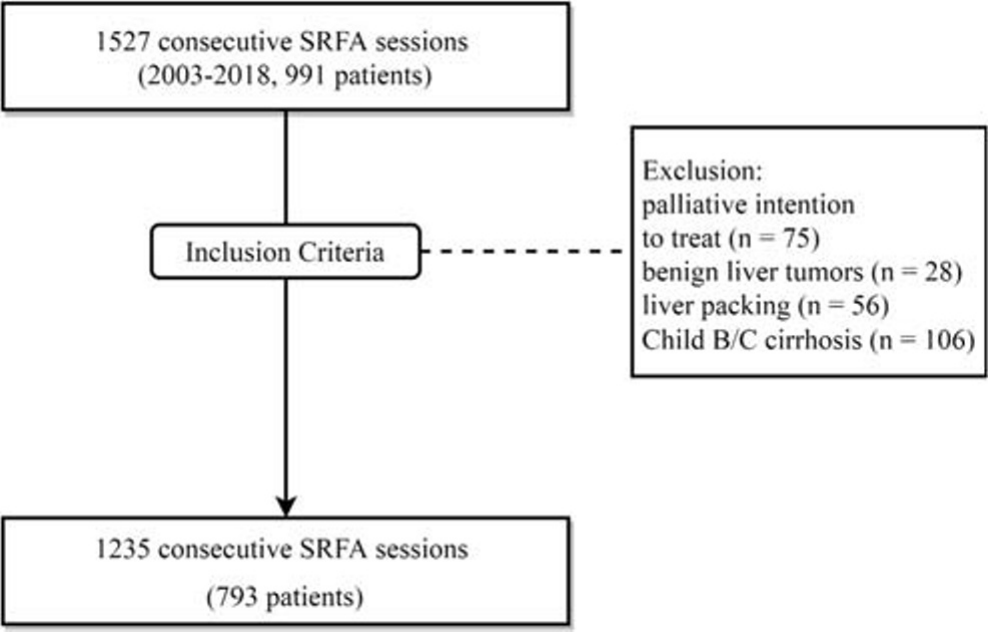
Flowchart of SRFA selection

This retrospective, single-center study was approved by the Institutional Review Board. Written informed consent for SFRA was obtained from the entire study population. All cases were reviewed, and treatment plans approved by consensus in multidisciplinary tumor board meetings consisting of hepatologists, oncologists, transplant surgeons, and interventional radiologists. Therapy decisions were made considering tumor and patient-specific characteristics such as size, number, anatomical location, and performance status. Owing to our stereotactic multi-probe approach, a potentially local curative treatment could be offered without limitations on tumor size or number. Thus, BCLC-B and BCLC-C patients were offered SRFA as first-line therapy despite BCLC guidelines recommending treatment with transarterial chemoembolization or medical therapy. Between July 2003 and December 2018, we performed 1527 consecutive SRFA sessions for primary and secondary liver tumors. Ablations for benign liver lesions (*n* = 28), those with palliative intention to treat due to extensive tumor spread (*n* = 75), liver packing prior to SRFA (*n* = 56), and ablations of patients with advanced liver cirrhosis (child B and C, *n* = 106) were excluded (Fig. 1). All other ablations were included in the study.

Tumor diagnosis was made using typical imaging appearances on multiphasic contrast MRI or CT in addition to histopathological confirmation before or during the SRFA procedure where imaging was inconclusive.

### Multi-probe stereotactic radiofrequency ablation

**Fig. 2:**
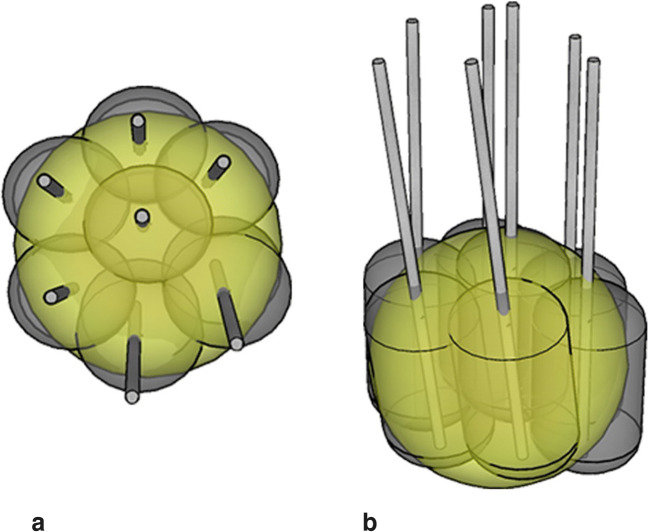
Illustration of multi-probe RF ablation with multiple overlapping ablation zones (grey ellipsoids with central RF probe) covering the entire tumor volume (yellow sphere); **a** top view, **b** oblique view

The basic principle of multi-probe RF ablation with intraoperative image fusion is to produce multiple overlapping ablation zones to entirely cover tumors with a sufficient safety margin (Fig. 2), allowing for larger tumor ablations than single ablation zones. Stereotactic technique relates the position of targets and entry points to a Cartesian coordinate system, and therefore allows for reliable planning and execution of interventional procedures, in this case tumor ablation.

### Stereotactic radiofrequency ablation procedure

The method of SRFA with image fusion for planning, intraoperative verification of coaxial needle placement, and ablation result has been previously described in detail [15]. In brief, the key steps are as follows:

I. Preparation: treatment is performed under general anesthesia with muscle paralysis and immobilization facilitated by a single (Bluebag, Medical Intelligence Inc.) or double vacuum fixation technique (BodyFix, Medical Intelligence Inc.); 10–15 broadly attached registration markers (Beekley Spots, Beekley Corporation) are applied to the skin for image-to-patient registration.

II. Planning: a contrast-enhanced CT (Siemens SOMATOM Sensation Open, 82-cm-diameter sliding gantry, Siemens AG) with a 3-mm slice interval is acquired in the axial plane in arterial and venous phases. The obtained CT data is then transferred to an optically based navigation system (Stealth Station Treon plus, Medtronic Inc.) to plan one or multiple trajectories using multiplanar and 3D reconstructed images.

III. Execution—co-axial needle placement: to eliminate respiratory motion, temporary disconnections of the endotracheal tube are performed during the planning CT, during each stereotactic needle placement, and for the final CT. After registration, an accuracy check and sterile draping, the ATLAS aiming device (Elekta PSC Medical Intelligence Inc.), is used for navigated trajectory alignment. 15G/17.2-cm coaxial needles (Bard Inc.) are placed through the aiming device (without real-time imaging) to house radiofrequency electrodes. An unenhanced CT is then performed for verification of correct needle placement and fused with the planning CT using the navigation system’s image 3D registration algorithm. A 16G biopsy sample is obtained via one of the coaxial needles for patients without prior histological confirmation.

III. Execution—RF ablation: a maximum of three 17G RF-electrodes (Cool-tip, Medtronic, 25 cm in length with 3 cm exposure) are introduced through the coaxial needles for serial tumor ablation. RF ablation is carried out using the unipolar Cool-tip_RF generator (Cool-tip, Medtronic), including the Cool-tip_RF switching controller. The standard ablation time for three electrodes is 16 min although if a significant increase in impedance (the so-called roll-off effect) is encountered sooner, the ablation process is considered complete. Track ablation is carried out during needle repositioning and final removal to prevent bleeding and potential tumor seeding.

IV. Final control CT: post-ablation contrast-enhanced CT is carried out in the arterial and portal venous phases and fused with the planning CT to assess ablation zone coverage and complications.

**Fig. 3:**
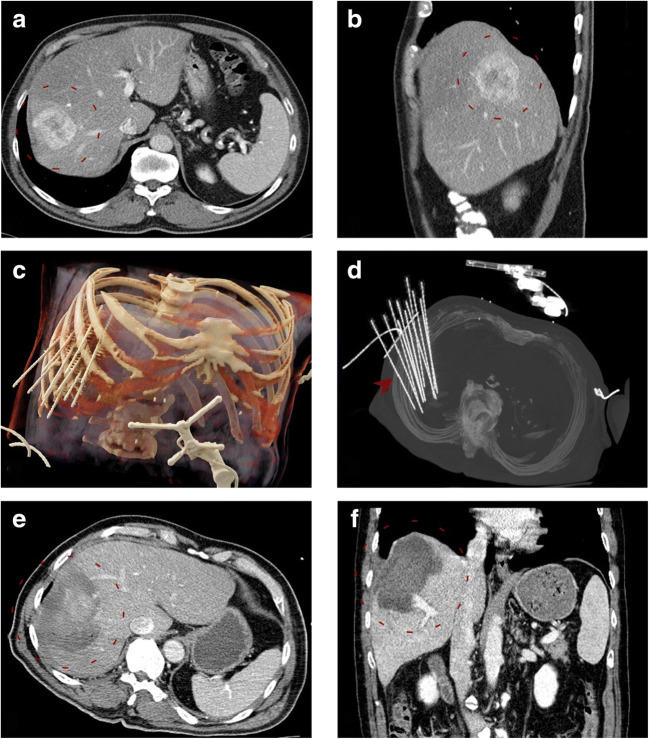
Case of an 81-year-old male with a single 6.0-cm HCC in segment VII/VIII. **a**, **b** Portal venous phase initial CT scan with a hyper-enhancing nodule in segment VII/VIII (*red dashed circle*). **c**, **d** Volume rendering and maximum intensity projection (MIP) of the native control CT with 11 needles in place (*red arrowhead*). **e** Portal venous phase final control CT scan showing complete ablation zone (*red dashed circle*). No complications were noted. **f** The *red dashed circle* illustrates the ablation zone at 6 months with no evidence of a late complication or local recurrence

Images from an example SRFA case are shown in Fig. 3.

### Adaptations over time

SRFA was introduced at our institution in 2003 and the following adaptations were made, in chronological order: (i) avoiding pulmonary transgression: 3D planning on multiplanar re-constructed images allows for angulated needle paths, which avoid pleural or pulmonary transgression; (ii) management of hemorrhage without hemodynamic compromise: 15-min manual external compression was used to treat acute bleeding detected in the final CT; (iii) long-term antibiotics in patients with bilioenteric anastomosis or other risk factors for ascending biliary infection were treated with at least 3 weeks of post-interventional antimicrobial drugs with good biliary penetration; and (iv) management of pleural effusions: pleural effusions were treated with chest drains only after failure of conservative measures.

### Complications

Complications were recorded at the time of SRFA and also evaluated using all available medical records including imaging reports. Major complications were defined according to the Society of Interventional Radiology (SIR) Standards of Practice Committee classification [16]. Perioperative mortality was defined as death occurring within 30 days after ablation.

### Definitions of risk factors

A distance of < 1 cm was defined as “close to” in terms of the diaphragm, liver capsule, organ, major vessel, central bile duct, or gall bladder. Major vessel was defined as a vessel with a diameter of > 3 mm. Non-critical location was defined as a location without adjacent vulnerable structures. History of bile duct treatment includes sphincteroplasty, papillotomy, and bilioenteric anastomosis.

### Statistical analysis

Statistical analysis was performed using IBM SPSS software version 20. Data were expressed as total numbers, median, and range.

The difference between categorical variables was evaluated with the *X*^2^ test, whereas the Mann-Whitney *U* test was used to evaluate independent continuous variables. Binary logistic regression was used to evaluate possible predictors of major complications (categorical outcome) where variables of interest identified in simple logistic regression analysis (*p* value < 0.1) were further analyzed in a multivariable model. A *p* value < 0.05 was considered as statistically significant.

## Results

### Patient characteristics (Table 1)

Seven hundred ninety-three patients, 241 (30.4%) females and 552 (69.6%) males, with a median age of 65.0 years (0.3–88.0) underwent SRFA for treatment of primary and secondary liver tumors. Diagnoses included 350 (44.1%) hepatocellular carcinomas (HCCs), 36 (4.5%) intrahepatic cholangiocarcinomas (ICCs), and 407 (51.3%) metastatic tumors, whereby the majority (218) were of colorectal origin. The median size of the 2475 nodules was 3.0 cm (0.5–19.0 cm). Patients had a median of 2 tumors (1–29) treated in 1235 sessions. At the beginning of treatment, 433 (54.7%) patients had a solitary tumor in the liver, 186 (23.5%) had two tumors, 83 (10.5%) had three tumors, and 91 (11.3%) had more than three tumors (defined as multiple tumors). Two hundred ninety-four (37.1%) patients had underlying cirrhosis.

**Table 1:** Patient characteristics of 793 patients undergoing 1235 SRFA sessions for 2475 tumors

Patient characteristics	
Age, years (range)	65.0 (0.3–88.0)
Sex (female/male), *n* (%)	241/552 (30.4/69.6)
Tumor type, *n* (%)	
HCC, *n* (%)	350 (44.1)
ICC, *n* (%)	36 (4.5)
Metastasis, *n* (%)	407 (51.3)
Colorectal, *n* (%)	218 (61.2)
Other, *n* (%)	189 (38.8)
Cirrhosis, *n* (%)	294 (37.1)
Number of tumors at initial SRFA	
*n* = 1, *n* (%)	433 (54.7)
*n* = 2, *n* (%)	186 (23.5)
*n* = 3, *n* (%)	83 (10.5)
*n* > 3, *n* (%)	91 (11.3)
Tumor size at initial SRFA	
< 3 cm, *n* (%)	382 (48.0)
3–5 cm, *n* (%)	275 (34.8)
> 5 cm, *n* (%)	136 (17.2)
At initial SRFA	
Tumors, median (range)	1 (1–9)
Tumor size, median (range)	3.0 cm (0.5–18.0)
Overall treated	
Tumors, median (range)	2 (1–29)
Tumor size, median (range)	1.9 (0.5–19.0)
Median no. of ablations per patient	1 (1–10)

*SRFA* stereotactic radiofrequency ablation, *HCC* hepatocellular carcinoma, *ICC* intrahepatic cholangiocarcinoma

### Perioperative mortality (Table 2)

Thirty-day mortality was 0.5% (6/1235) with four deaths occurring from major hemorrhage, one due to sepsis, and one as a consequence of acute-on-chronic renal failure.

**Table 2:** Details of perioperative deaths

ID	Age	Sex	Prim.	Cirr.	T/S	mS/S	N/S	Fatal complication	Therapy
**1**	77	Male	CRC	–	3	5.5 cm	12	Major hemorrhage, hemorrhagic shock (0 days)	AG-coiling, ICU
**2**	83	Female	HCC	–	3	4.8 cm	12	Major hemorrhage, MODS (19 days)	AG-coiling, ICU
**3**	73	Male	HCC	Child A	1	5.5 cm	3	Major hemorrhage, MODS (20 days)	AG-coiling, ICU
**4**	75	Male	ICC	–	1	8.0 cm	6	Major hemorrhage, MODS (18 days)	AG-coiling, ICU
**5**	79	Male	CRC	–	1	6.5 cm	9	Liver abscess, septic shock (21 days)	Surgery, ICU
**6**	58	Male	HCC	Child A	3	1.5 cm	4	Acute on chronic renal failure (24 days)	ICU

*N/S* needles per session, *HCC* hepatocellular carcinoma, *ICC* intrahepatic cholangiocarcinoma, *CRC* colorectal carcinoma, *Cirr*. cirrhosis, *AG* angiography, *US* ultrasound

### Major complications

**Fig. 4:**
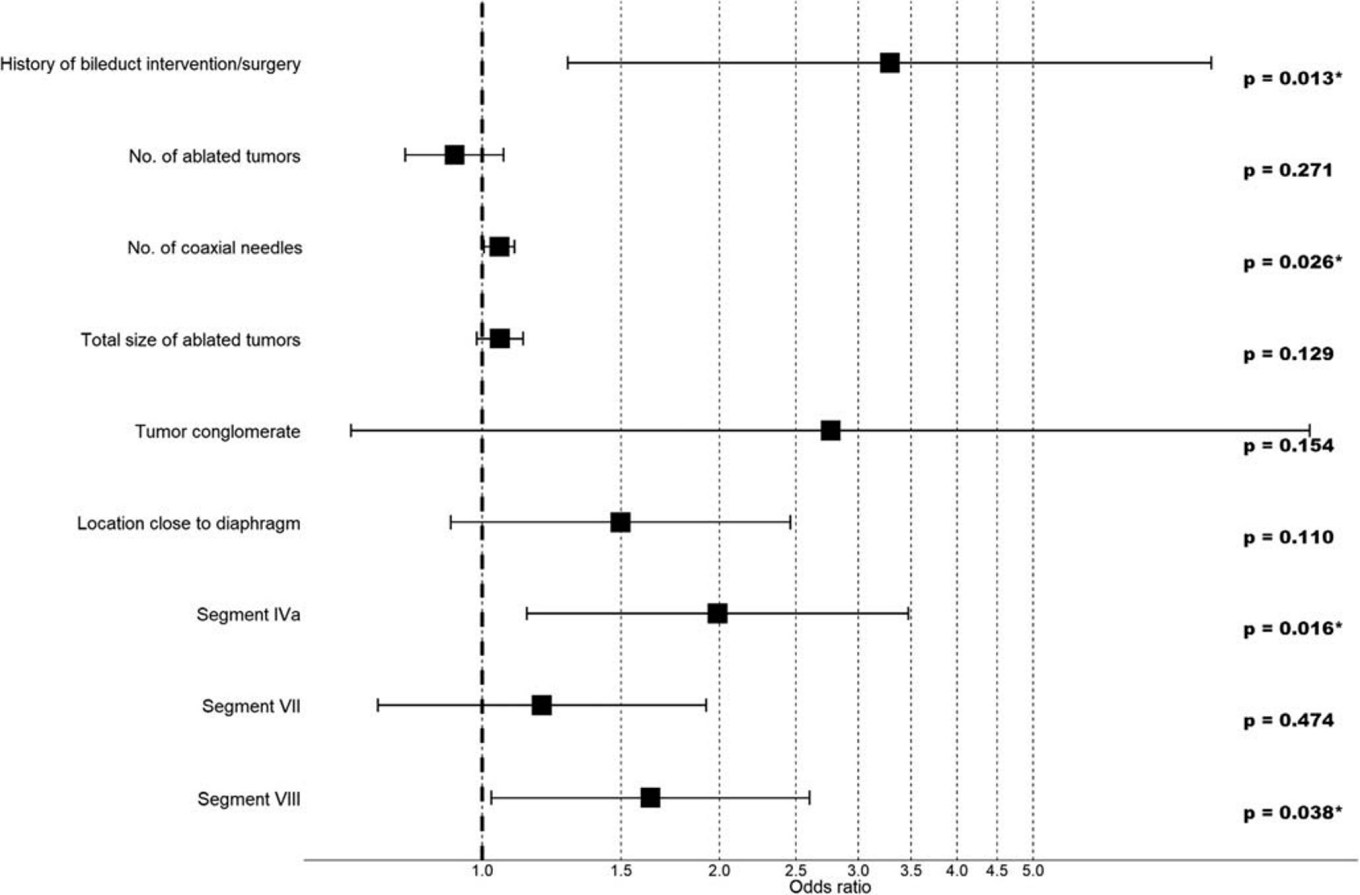
Forest plot of multivariable logistic regression analysis of major complications

Major perioperative complications are shown in Table 3, where the major complication rate was 7.4% (91 of 1235 ablation sessions). In addition to the aforementioned fatal complications, in descending order of severity, transient liver failure (1 patient), transient respiratory failure (5 patients), and transient cardiac problems (3 patients) required intensive care unit admission (Fig. 4). Thermal damage to hollow viscera (3 patients) and the skin (2 patients) had to be surgically repaired. Thermal injury to central bile ducts required ERCP and US-guided drainage. One case of thermal injury along the needle track resulted in a pleuro-cutaneous fistula which required repeated US-guided drainages. Eleven liver abscesses, with bilio-pleural fistulation in two cases, required surgical (6 patients) or interventional (US-guided drainages, 5 patients) treatment. 54.5% of the patients with abscesses had a positive history of bile duct surgery or intervention. One arterio-portal fistula was treated with angiographic coiling. Other complications included peri-/intrahepatic hemorrhage (30 patients) requiring angiographic coiling, major pleural effusions (11 patients) requiring thoracocentesis, and pneumothorax (16 patients) requiring chest tube insertion.

**Table 3:** Major complications after SRFA

Complication	No.	Therapy	Treated in same GA session
Hemorrhagic shock	4	AG-coiling, ICU, death (4)	–
Septic shock, liver abscess (1)	1	ICU, surgery, drainage, death (1)	–
Acute on chronic renal failure	1	ICU, death (1)	–
Transient liver failure	1	ICU	–
Transient respiratory failure	5	ICU	–
Transient cardiac problems	3	ICU	–
Thermal injury of hollow visceral organs	3	Surgery	–
Thermal injury of the skin	2	Surgery	–
Thermal injury of the bile duct	1	ERCP	–
Thermal injury of the pleura and skin with fistulation	1	repeated US drainages	–
Liver abscess, w. bilio-pleural fistula (2)	11	Surgery (6), US-guided drainage (5)	–
Pleural effusion	11	Thoracentesis	–
Arterio-portal fistula	1	AG-coiling	–
Pneumothorax	16	Thoracostomy tube	17
Peri-/intrahepatic hemorrhage	30	AG-Coiling	34

*SRFA* stereotactic radiofrequency ablation, *No*. number, *ICU* intensive care unit, *AG* angiography, *US* ultrasound, GA general anesthesia

## 50.5% (46/91) of all major complications were successfully treated in the same anesthetic session by angiographic coiling for hemorrhage and chest tube insertion for pneumothorax.

### Risk factors for major complications

Simple logistic regression analysis showed that significant predictors of major complications were (i) history of bile duct surgery or intervention (*p* = 0.021), (ii) number of coaxial needles (for RF probe positioning) (*p* < 0.001), (iii) number of ablated tumors (*p* = 0.030), (iv) total tumor size per ablation session (*p* < 0.001), (v) location close to the diaphragm (*p* = 0.010), (vi) tumor conglomerate (*p* = 0.022), and (vii) location in segment IVa (*p* = 0.021), (viii) segment VII (*p* = 0.018), and (ix) segment VIII (*p* < 0.001). After multivariable analysis, history of bile duct surgery/intervention (*p* = 0.013, OR = 3.290), number of coaxial needles (*p* = 0.026, OR = 1.052), and location of tumors in segment IVa (*p* = 0.016.013, OR = 1.989) or VIII (*p* = 0.038, OR = 1.635) were found to be independent prognostic factors (Table 4, Fig. 4).

**Table 4:** Uni- and multivariable logistic regression analyses of risk factors for major complications after a learning curve

Variables	No. of sessions	Major compl. (%)	Simple A. *p* value	Multivariable analysis		
				*p* value	Odds ratio	95% CI
Age (> 70/< 70)	386/849	8.5/6.8	0.29	–	–	–
Sex (female/male)	350/885	8.3/7.0	0.44	–	–	–
Tumor type (HCC/ICC/metastatic)	538/86/ 611	6.1/7.0/8.5	0.13	–	–	–
Liver cirrhosis (+/−)	441/794	5.9/8.2	0.14	–	–	–
No. of needles	1235	–	< 0.001*	0.026*	1.052	1.006–1.099
No. of ablated tumors	1235	–	0.030*	0.271	0.923	0.799–1.065
Total size of ablated tumors	1235	–	< 0.001*	0.129	1.054	0.985–1.127
BD treatment^±^	33/1202	18.2/7.1	0.021*	0.013*	3.290	1.285–8.421
Location close to						
Diaphragm	250/985	11.2/6.4	0.010*	0.110	1.499	0.913–2.461
Liver capsule	581/654	8.6/6.3	0.12	–	–	–
Organ	188/1047	7.4/7.4	0.96	–	–	–
Major vessel	361/874	8.6/6.9	0.52	–	–	–
Central bile duct	41/1194	12.2/7.2	0.24	–	–	–
Gall bladder	46/1189	8.7/7.3	0.73	–	–	–
Non-critical location	454/781	7.0/7.6	0.74	–	–	–
Conglomerate	11/1224	27.3/7.2	0.022*	0.154	2.767	0.682–11.224
Liver segments				–	–	–
Segment I	81/1154	11.1/7.1	0.19	–	–	–
Segment II	224/1011	4.9/7.9	0.12	–	–	–
Segment III	198/1037	8.1/7.2	0.68	–	–	–
Segment IVa	160/1075	11.9/6.7	0.021*	0.016*	1.989	1.139–3.470
Segment IVb	93/1142	7.5/7.4	0.95	–	–	–
Segment V	266/969	6.0/7.7	0.34	–	–	–
Segment VI	355/880	8.5/6.9	0.36	–	–	–
Segment VII	466/769	9.7/6.0	0.018*	0.474	1.191	0.738–1.924
Segment VIII	504/731	10.5/5.2	< 0.001*	0.038*	1.635	1.028–2.601

*HCC* hepatocellular carcinoma, *ICC* intrahepatic cholangiocarcinoma, *No*. number, *Compl*. complication

^±^Includes sphincteroplasty, papillotomy

*Statistically significant

**Fig. 5:**
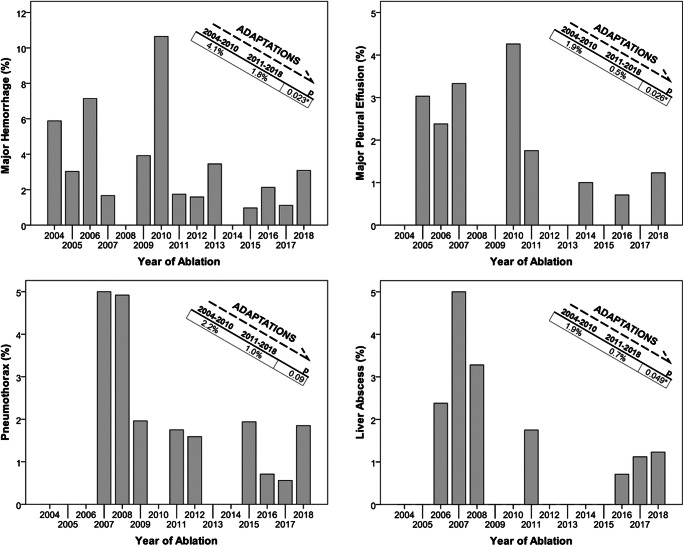
Overview of the most frequent major complications between 2004 and 2018

The median summated size of treated tumors and number of coaxial needles (for RF probe positioning) was 6.2 cm (0.5–23) and 9 (1–33), respectively, in sessions associated with complications vs. 3.7 cm (0.5–31) and 6 (1–38) for those without complications whereby differences reached statistical significance (*p* < 0.001 for both comparisons). The median ablation time per session was 32 min (10–330 min).

With regard to liver abscesses as one of the most serious complications, a positive history of bile duct surgery or interventions showed a significant impact on their occurrence: Liver abscesses occurred significantly more frequently in patients with bile duct history with 18.2% (6/33) vs. 0.5% (6/1202) (*p* = 0.000).

Fever (> 37 °C, measured peripherally) developed in all patients but subsided in 1–2 days with symptomatic treatment. Median postoperative hospital stay was 4 days (1–42) and significantly longer in patients who developed major complications with median 7.5 days (3–42) (*p* < 0.001).

### Adaptations over time (Fig. 5)

Several adaptations, such as sparing of the pleural recess, external compression in case of post-procedural hemorrhage without hemodynamic compromise, more conservative management of pleural effusions, and long-term antibiotic treatment in case of pneumobilia or bile duct surgery, lead to an overall decrease of the major complication rate from 11.5% before January 2011 to 6.0% (36/314, 55/921, *p* = 0.001). Rates of the most common complications, namely pneumothorax, major hemorrhage, pleural effusion, and liver abscesses, decreased from 2.2 to 1% (7/314, 9/921, *p* = 0.09), 4.1 to 1.8% (13/314, 17/921, *p* = 0.023), 1.9 to 0.5% (6/314, 5/921, *p* = 0.026), and 1.9 to 0.7% (6/314, 6/921, *p* = 0.049), respectively.

### Efficacy and overall survival

The overall complete ablation rate in the present study was 96.2% (2382/2475 tumors). Based on tumor type, the complete ablation rate was 96.2% (957/995) for HCC, 94.5% (137/145) for ICC, and 95.5% (1288/1335) for liver metastasis. The corresponding overall local recurrence rate was 8.3% (205/2475) and 7.2% (72/995) for HCC, 13.8% (20/145) for ICC, and 8.5% (113/1335) for liver metastasis.

The overall survival (OS) rate for the entire study cohort was 89%, 60%, and 44% at 1 year, 3 years, and 5 years, respectively, with a median OS of 50 months (95 CI 43.9–56.4). Based on tumor type, the OS rates at 1 year, 3 years, and 5 years were 93%, 69%, and 53% for HCC; 82%, 51%, and 43% for ICC; and 86%, 53%, and 36% for liver metastasis.

## Discussion

In this series of 1235 ablation sessions over 15 years, we have shown that stereotactic radiofrequency ablation (SRFA) provides low levels of morbidity and mortality in the treatment of primary and metastatic liver tumors with a mortality of 0.5% (6/1235) and an overall major complication rate of 7.4% (91/1235). Furthermore, we found the major complication rate decreased from 11.5 (36/314) before January 2011 to 6.0% (55/921) after January 2011 which could be attributable to procedural adaptations, increasing experience, and improved patient selection.

Major complication rates in the present study are similar to those quoted in the conventional tumor ablation literature (i.e., without navigation systems) of 0.9–10.0% [4, 8, 9], despite our selection of large (17% > 5 cm) and multiple tumors (21.8% *n* ≥ 3). This observation could reflect the additional safety that is offered by stereotactic guidance through careful planning and precision targeting.

The only independent predictive factors for major complications were history of bile duct surgery/intervention, number of coaxial needles, and location of tumors in segment IVa or VIII. Total tumor size was shown to be predictive of major complications at simple logistic regression analysis, although it was not an independent predictive factor at multivariable analysis.

In the present study, we have excluded patients with deteriorated liver function (child B and C cirrhosis) because the associated major complication of liver failure after treatment cannot be avoided by the adaptation or improvement of treatment technique. This is in contrast to technique-related complications such as hemorrhage, pleural effusion, or pneumothorax; their occurrence can be reduced or even prevented by technical improvements of SRFA compared to conventional RFA.

The historic literature suggests that percutaneous RFA of liver tumors in difficult locations leads to more complications, insufficient ablation, and poor tumor control [17, 18], particularly in the case of subcapsular lesions [14, 17, 19, 20]. However, more recently, studies have reported similar complication rates for subcardiac vs. non-subcardiac tumors (1–7.7%) [21–23]. In line with these reports, we found that subcapsular tumors were not associated with higher major complication rates following logistic regression analysis (*p* = 0.12). Lesions high in the liver require either transthoracic [20, 24, 25] or steeply angulated transhepatic approaches [26], with some investigators using adjunctive hydrodissection techniques [27]. Lower major complication rates of 10% (6/60) have been reported using transhepatic approaches [28] vs. 57.9% for transpleural approaches [29], which is why we favor the former. However, angulated multiprobe ablations pose severe technical challenges which in practice limits the size of lesions that can be treated without stereotactic guidance. While laparoscopic RFA can also be used to treat difficult lesions, it is more invasive with higher rates of reported major complications at 10–12.4% [28, 30]. Surgical resection is associated with even higher major complication rates at 10–27% for laparoscopic and 18–37% for open resection [31–35]. Simple logistic regression analysis showed that lesions close to the diaphragm at the hepatic dome were a predictor of major complication (11.2% vs. 6.4%); however, this was not found to be an independent risk factor at multivariable analysis.

Furthermore, 62.6% of major complications including pleural effusion, pneumothorax, and major hemorrhage could be easily managed with a single interventional procedure and 50.5% were managed in the same general anesthetic session without changing the postoperative course.

Several adaptations resulted in a significant decrease in major complication rate from 11.5% before January 2011 to 6.0% (*p* = 0.001). Specifically, sparing of the pleural recess resulted in a decrease in pneumothorax from 2.2 to 1.0% (*p* = 0.09). However, the risk for pneumothorax remains, since lesions located in the liver dome may require paths through the pleural recess. Post-procedural (usually perihepatic) hemorrhage without hemodynamic compromise was managed with manual external compression, which reduced the rate of subsequent angiographic coiling from 4.1 to 1.8% (*p* = 0.023). A more conservative, non-inferior, management of pleural effusions leads to a decrease in thoracentesis from 1.9 to 0.5% (*p* = 0.026). In case of pneumobilia or bile duct surgery, long-time antibiotic treatment resulted in a decreased rate of liver abscesses from 1.9 to 0.7% (*p* = 0.049).

The limitations of our study include its retrospective design and single treatment center bias, which reduces the generalizability of our data. Limited use of navigation systems as part of ablation procedures also reduces the generalizability, although use is gaining popularity.

In conclusion, stereotactic radiofrequency ablation (SRFA) of liver tumors is safe and can extend the treatment spectrum of conventional RFA, whereby history of bile duct surgery/intervention, number of coaxial needles, and location of tumors in segment IVa or VIII are the statistically most important risk factors for major complications. Adaptations over time with increasing experience lead to a significant decrease in complications.

## Abbreviations

HCC: Hepatocellular carcinoma
HR: Hepatic resection
ICC: Intrahepatic cholangiocarcinoma
ICU: Intensive care unit
RF: Radiofrequency
SRFA: Stereotactic radiofrequency ablation
US: Ultrasound
